# Supplementary material for: Operando real-space imaging of a structural phase transformation in the high-voltage electrode LixNi0.5Mn1.5O4
Source: Nat Commun. 2024 Dec 30;15:10783. doi: 10.1038/s41467-024-55010-6 (PMC11685944; doi:10.1038/s41467-024-55010-6)
Supplement: Supplementary file 1 — Supplementary Information [file 41467_2024_55010_MOESM1_ESM.pdf]

Operando real-space imaging of a structural phase transformation in the high-voltage electrode $Li_xNi_{0.5}Mn_{1.5}O_4$: Supplementary Information

Yifei Sun[1], Sunny Hy[2], Nelson Hua[3,4], James Wingert[3], Ross Harder[5], Ying Shirley Meng[2,6], Oleg Shpyrko[3], Andrej Singer[1,]*

[1]*Department of Materials Science and Engineering, Cornell University, Ithaca, New York 14850, USA*
[2]*Department of Nanoengineering, University of California San Diego, La Jolla, California, 92093, USA*
[3]*Department of Physics, University of California San Diego, La Jolla, California, 92093, USA*
[4]*PSI Center for Photon Science, Paul Scherrer Institute, 5232 Villigen PSI, Switzerland*
[5]*Advanced Photon Source, Argonne National Laboratory, Argonne, Illinois, 60439, USA*
[6]*Pritzker School of Molecular Engineering, University of Chicago, Chicago, Illinois, 60637, USA*
[*]asinger@cornell.edu

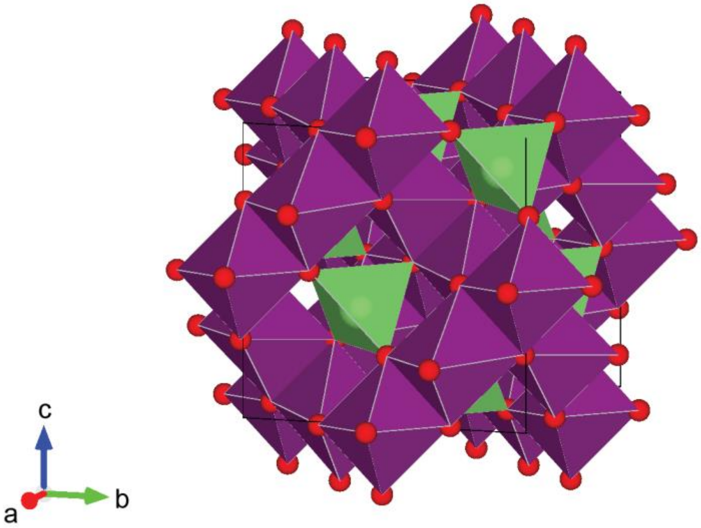

Supplementary Fig. 1. Crystal structure of the disordered $Li_xNi_{0.5}Mn_{1.5}O_4$. The disordered $Li_xNi_{0.5}Mn_{1.5}O_4$ has the cubic spinel structure with the space group $Fd\bar{3}m$. The Ni and Mn reside within the purple octahedra with Ni occupying 25% of the sites and Mn occupying 75% of the sites randomly. The Li resides within the green tetrahedra. The red atoms are oxygens.

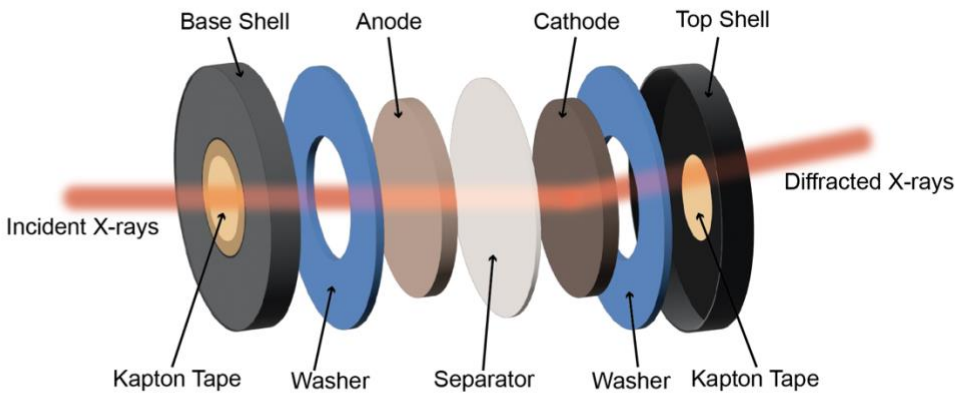

Supplementary Fig. 2. Expanded view of the operando coin cell. The cell top and base are from the standard CR2032 cells, which have a diameter of 20 mm and a height of 3.2 mm. Both sides have a hole drilled at the center of size around 3 mm in diameter, which is sealed by Kapton tape. The cell is placed so that the material of interest, the $Li_xNi_{0.5}Mn_{1.5}O_4$ cathode nanoparticles, is located downstream from the incident X-rays. The anode is lithium metal, and the separator (Celgard C480) contains electrolyte of 1 M solution of lithium hexafluorophosphate ($LiPF_6$) in a 1:1 volume mixture of ethylene carbonate (EC) and dimethyl carbonate (DMC).

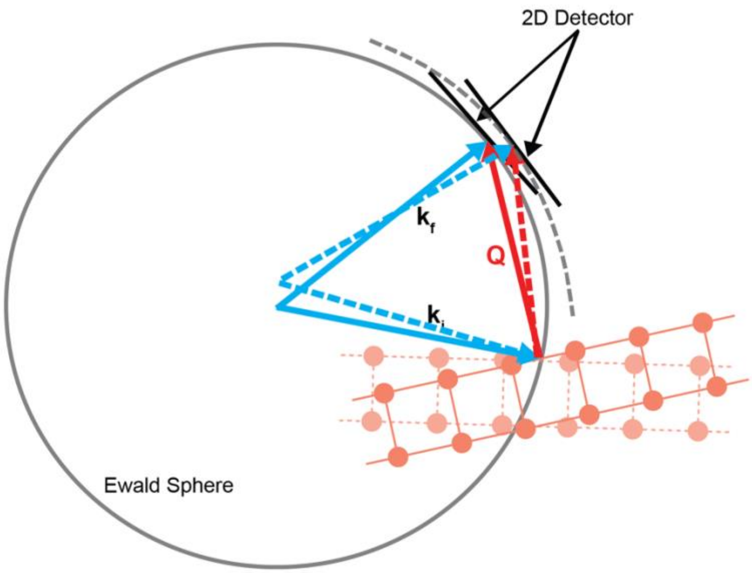

Supplementary Fig. 3. Ewald sphere construction. Rocking the crystal, shown as a lattice plane in red, results in the Ewald sphere slicing the vicinity of the Bragg peaks at different positions in the reciprocal space. This is equivalent to shifting the detector perpendicular to the Ewald sphere around the Bragg peaks. The nearly parallel slices were combined to obtain a 3D diffraction pattern[1].

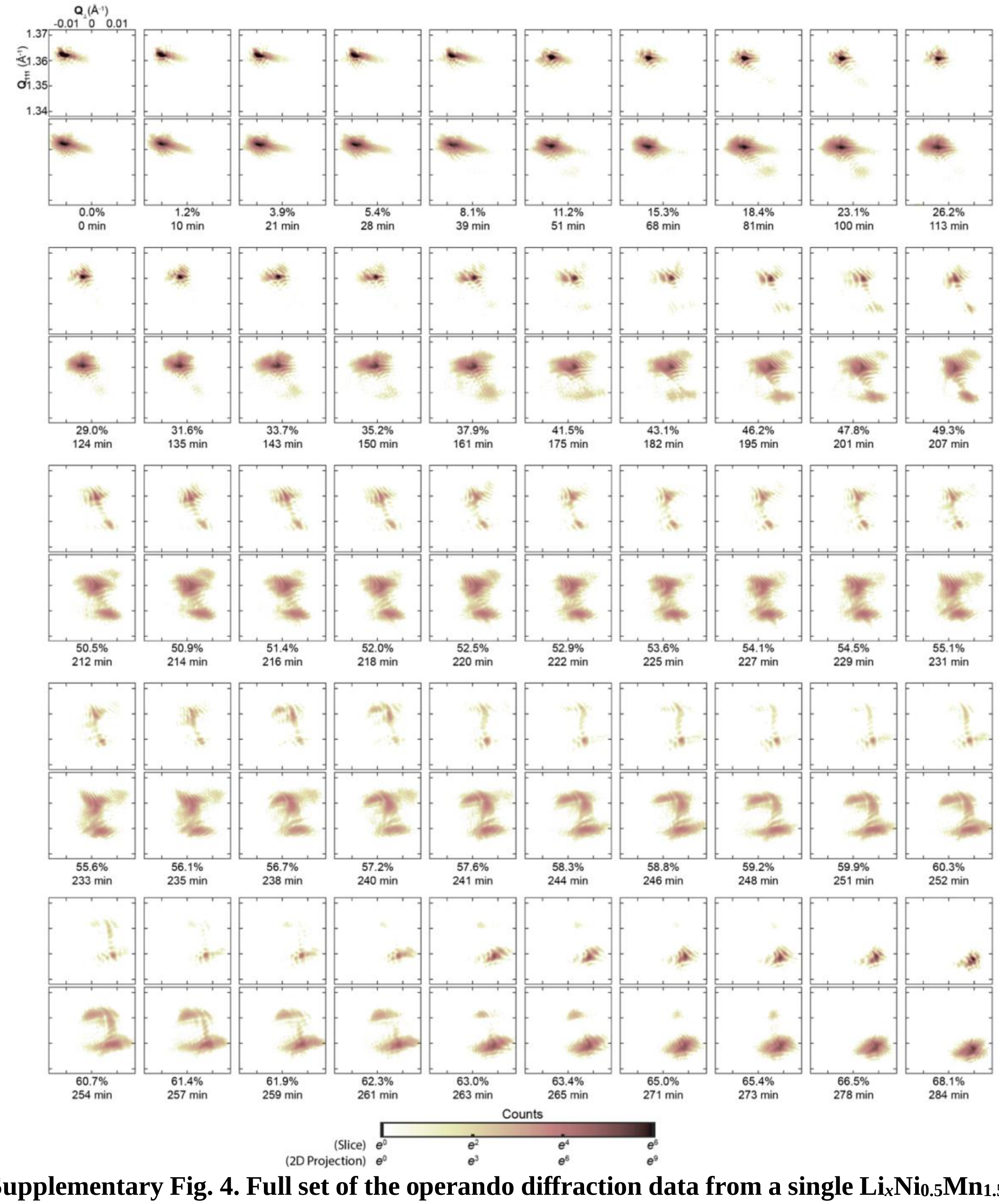

Supplementary Fig. 4. Full set of the operando diffraction data from a single $Li_xNi_{0.5}Mn_{1.5}O_4$ particle undergoing a structural phase transformation during discharge. (Top) 2D slice at the center of the 3D diffraction. (Bottom) 2D projection of the 3D diffraction. The diffraction data shows the decrease in intensity of the lithium-poor phase (larger Q) and the increase in intensity of the lithium-rich phase (smaller Q). During the transition, two peaks coexist, indicating the presence of both phases inside the $Li_xNi_{0.5}Mn_{1.5}O_4$ nanoparticle. The two-phase reaction ends around when the battery is 68% discharged. The diffraction data is inverted using the correlated phase retrieval algorithm.

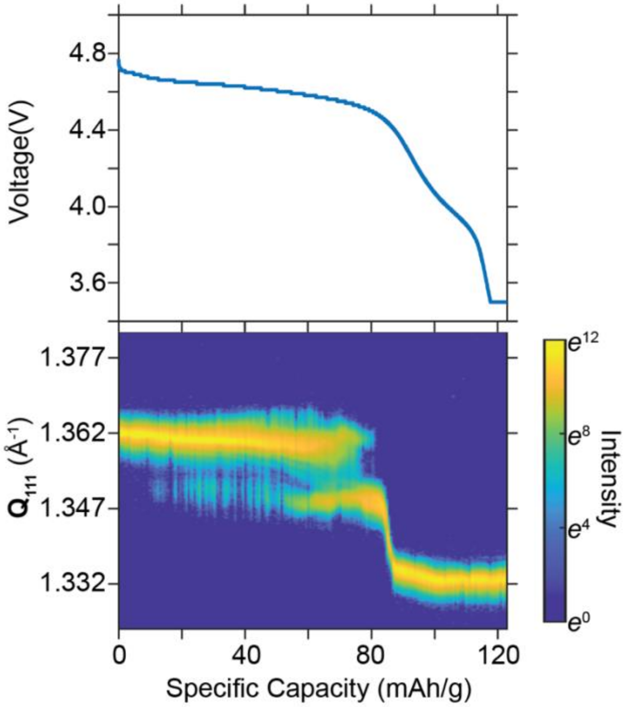

Supplementary Fig. 5. Electrochemical data of the cell (top) and diffraction data of a single $Li_xNi_{0.5}Mn_{1.5}O_4$ particle within the cell (bottom). The diffraction is calculated by collapsing the data in Fig. S4 into one dimension and shows good agreement with Fig. S4, where the 3D diffraction data is projected in 2D. The voltage plateau in the electrochemical data coincides with the two-peak coexistence region in the diffraction data. This indicates that the electrochemistry behavior of the $Li_xNi_{0.5}Mn_{1.5}O_4$ particle that we selected for reconstruction is representative of all active materials. When the specific capacity reaches around 80 mAh/g, the cell enters the solid-solution regime, which is reflected as one diffraction peak continuously shifting its Q position.

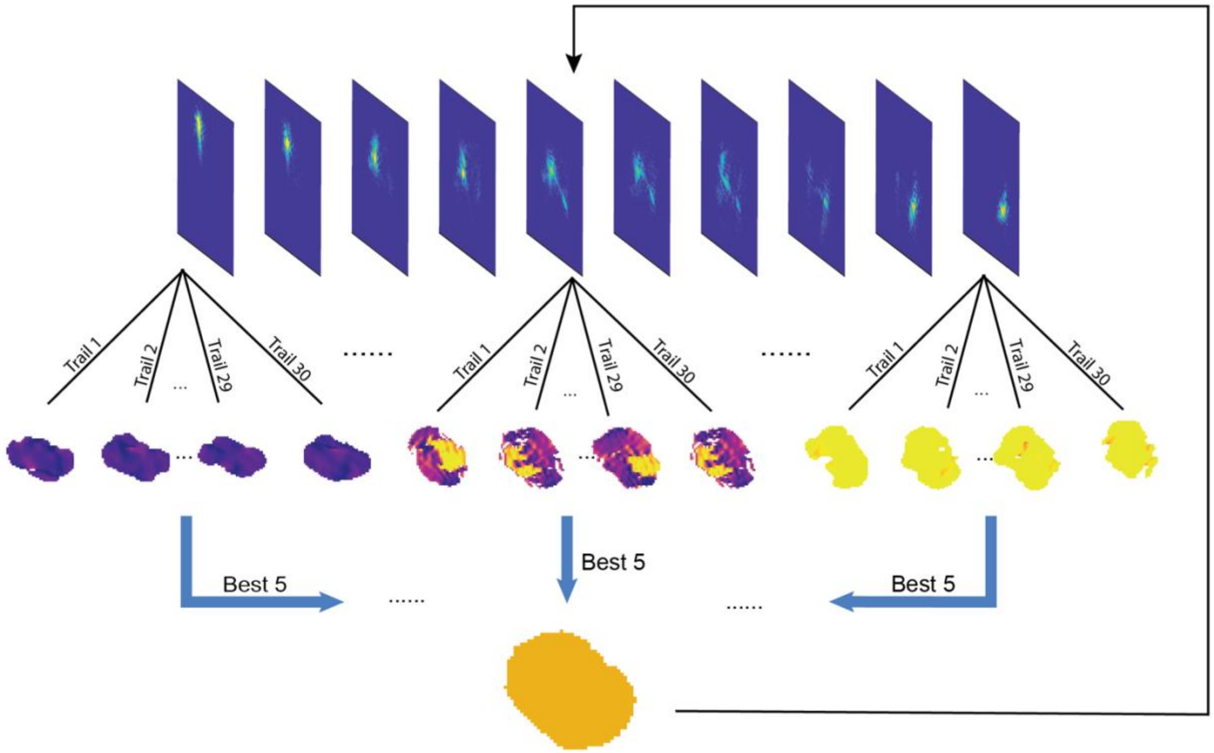

Supplementary Fig. 6. Illustration of the correlated phase retrieval algorithm. The illustration shows one iteration of the algorithm. A set of scans (we used 10 scans) that include both single-peak and two-peak diffraction are reconstructed with alternating Error Reduction (ER)[2] and Relaxed Averaged Alternating Reflections (RAAR)[3] phase retrieval algorithms separately 30 times. Each individual reconstruction is called a trail. Out of the 30 trails for each scan, we select the best 5 trails. The support of the reconstruction for the next iteration is then calculated by averaging the shape of the best 5 trails for all 10 scans. During the selection of the best 5 trails, the particle shape can appear inverted (two solutions, $S(\boldsymbol{r})$ and $S^*(-\boldsymbol{r})$, are indistinguishable, where $\boldsymbol{r}$ is the coordinate and * denotes complex conjugate). To determine if the reconstruction is inverted, we cross-correlate strain among different reconstructions.

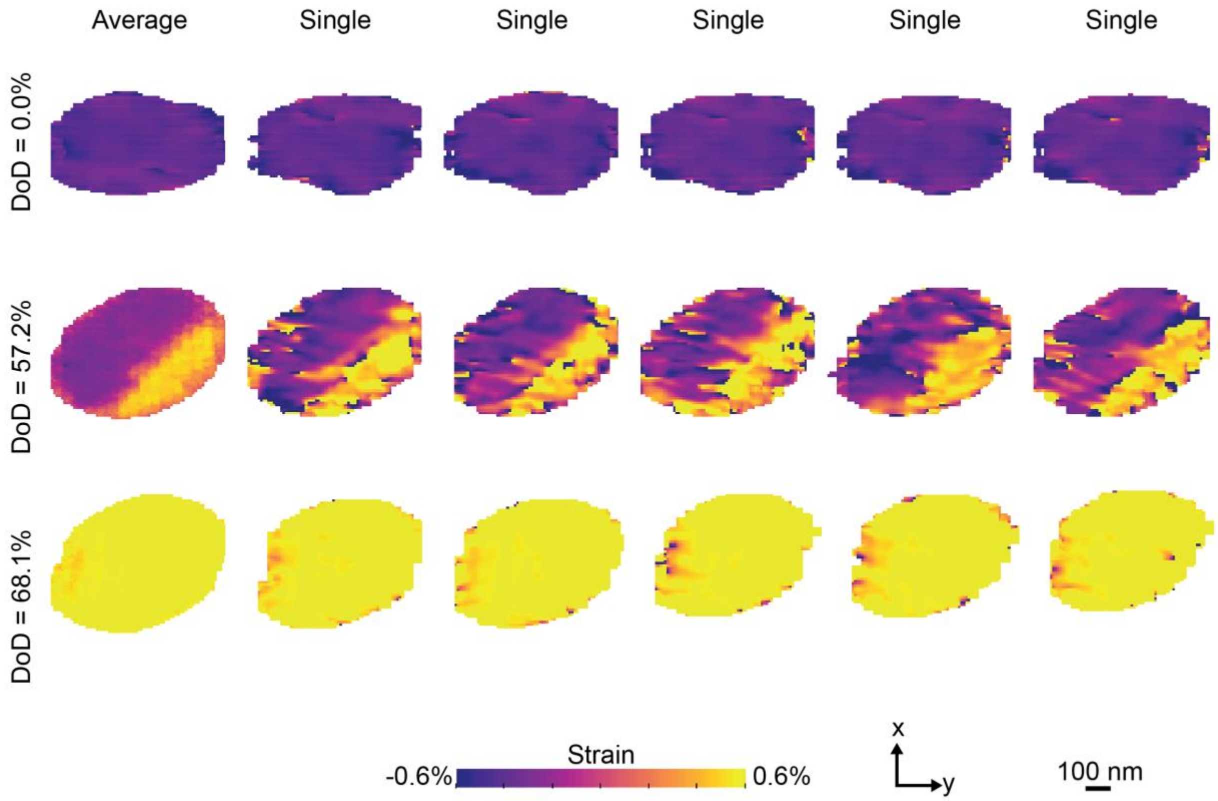

Supplementary Fig. 7. Comparison of strain between separate reconstructions and the average result. Each single reconstruction starts with a different random start. Strain is then calculated by taking the derivative of the reconstructed displacement field along the scattering vector Q. The average strain is taken from averaging individual strains of 50 reconstructions, each with a different random start. At different depths of discharge, 0%, 57.2%, and 68.1%, the strain of single reconstructions is consistent with each other, and the average strain is representative of the single strains.

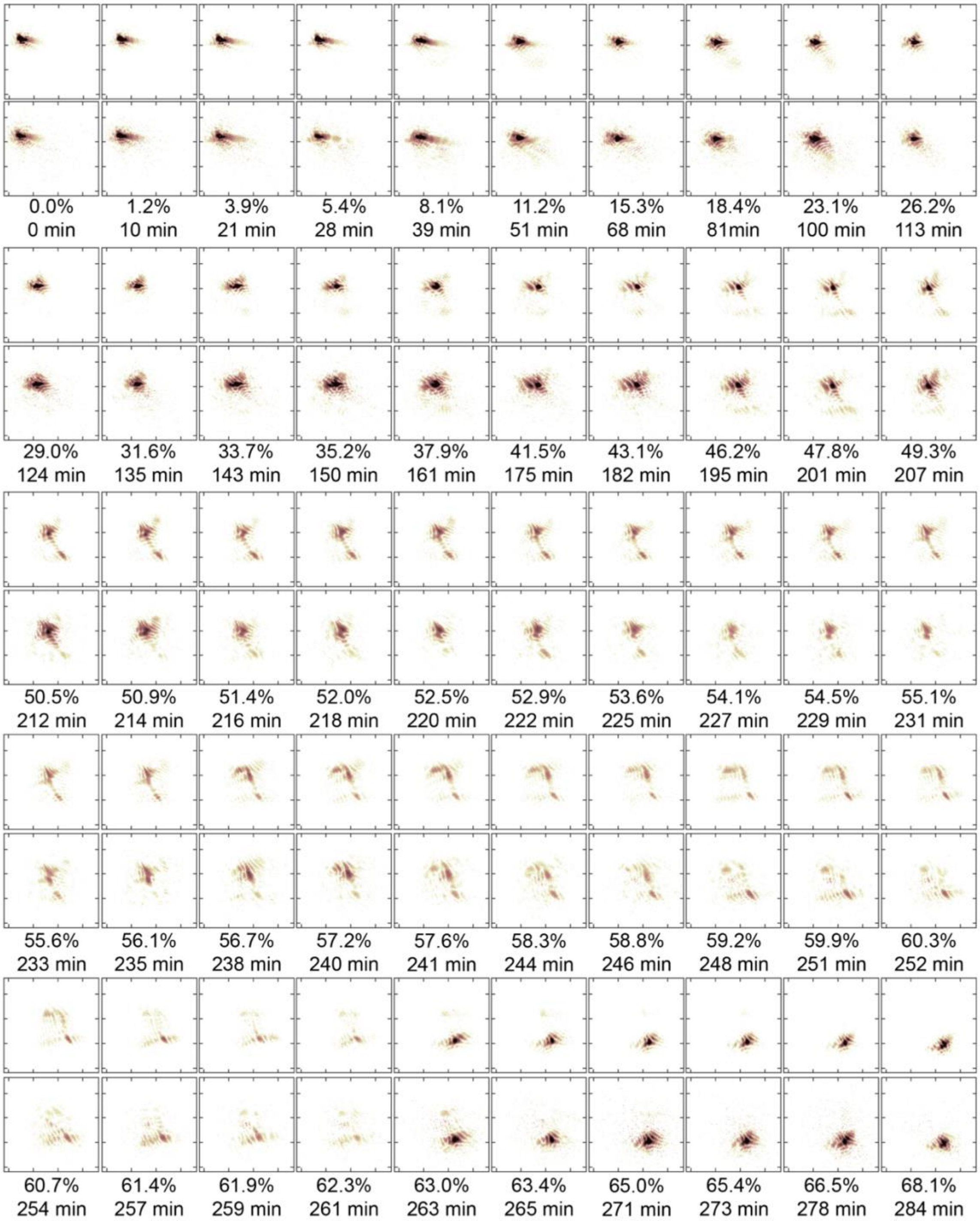

Supplementary Fig. 8. A side-by-side comparison of the central theta slice from the measured diffraction (top) and the Fourier transform of reconstructions (bottom). The close alignment between the reconstructed results and the diffraction data is indicative of successful phase retrieval. The false colors are identical to Fig. S4. Note that the diffraction pattern calculated from the reconstructed model shows a stronger interference fringe intensity than the measured diffraction data. This is because the computed shape has sharper boundaries compared to the shape of the real particle.

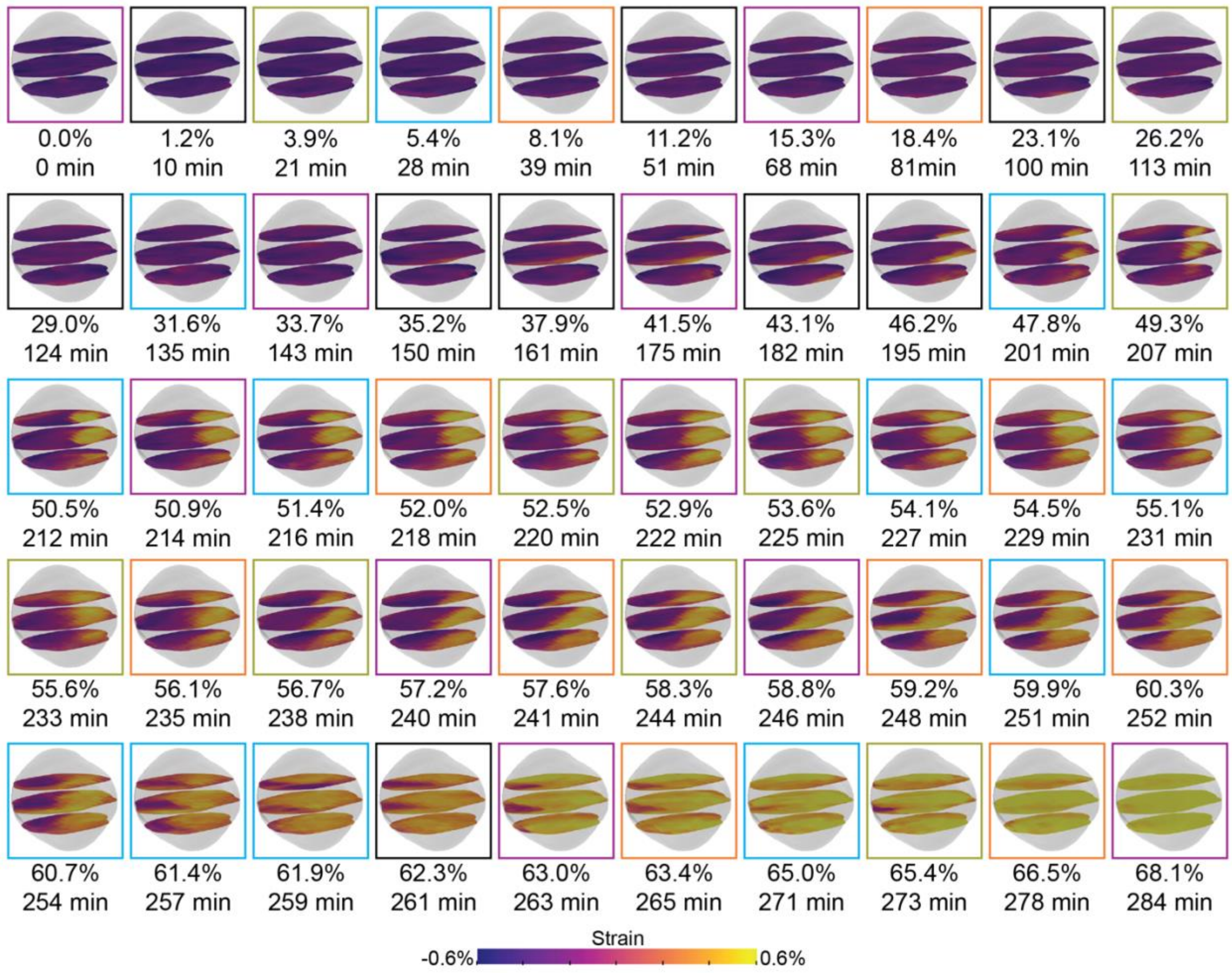

Supplementary Fig. 9. Full set of the operando imaging of the structural phase transformation during discharge. For each depth of discharge, the chosen planes at the same position are imaged for the visualization of strain distribution inside the entire particle. The color of the box around each image highlights different reconstruction runs. The particle starts with a uniform negative strain (Li-poor phase) and ends the two-phase reaction with a uniform positive strain (Li-rich phase). The intermediate stages show that the particle has both the red and blue phases, where the red phase grows at the expanse of the blue phase, defining a nucleation and growth mechanism for the two-phase reaction. The particle is about 500 nm large.

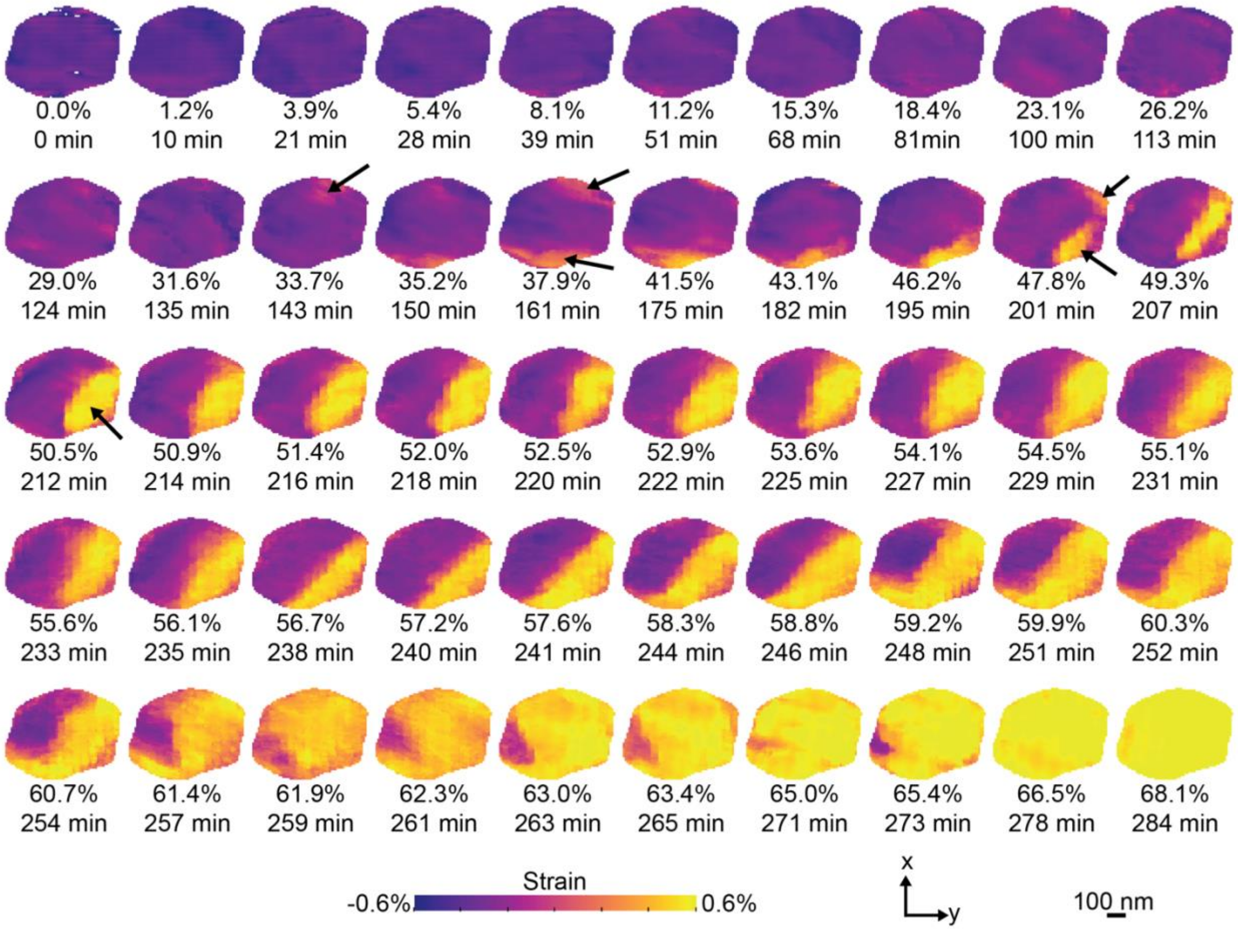

Supplementary Fig. 10. 2D view of the central slice in Fig. S9. The arrows indicate the nucleation sites of the new Li-rich phase (red) inside the Li-poor phase (blue). When the Li-rich phase nucleates, it starts around the edge of the particle and can occur simultaneously at multiple locations (DoD = 37.9%). As the Li-rich phase continues to grow, the nucleated sites grow and, in the meantime, coalesce (DoD =47.8%). Later, the nucleated sites merge for a total reduction of interface area (DoD = 50.5%).

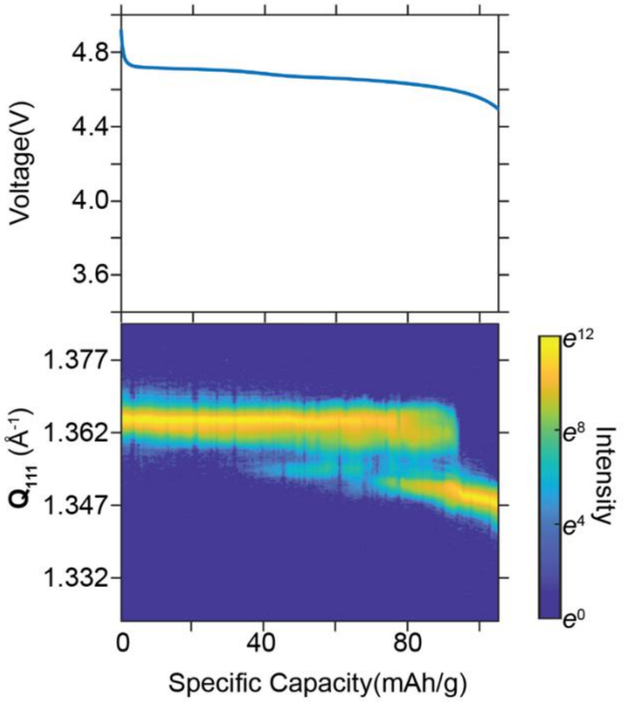

Supplementary Fig. 11. Electrochemical data of another cell (top) and diffraction data of another $Li_xNi_{0.5}Mn_{1.5}O_4$ particle in the cell (bottom) that was discharged at C/10 (18 mA/g). Similar to the particle discussed above, this supplementary particle also displays an extended period of two-phase coexistence during the discharge, as indicated by the peak splitting in the diffraction data. The two-phase region aligns with the voltage plateau in the electrochemical data. Compared to the primary particle investigated in the main text and Fig. S5, the Li-rich phase of the supplementary particle displays a slightly different q value. This difference arises because particles within the cells can undergo subtly different lattice dynamics during the phase transformation[4].

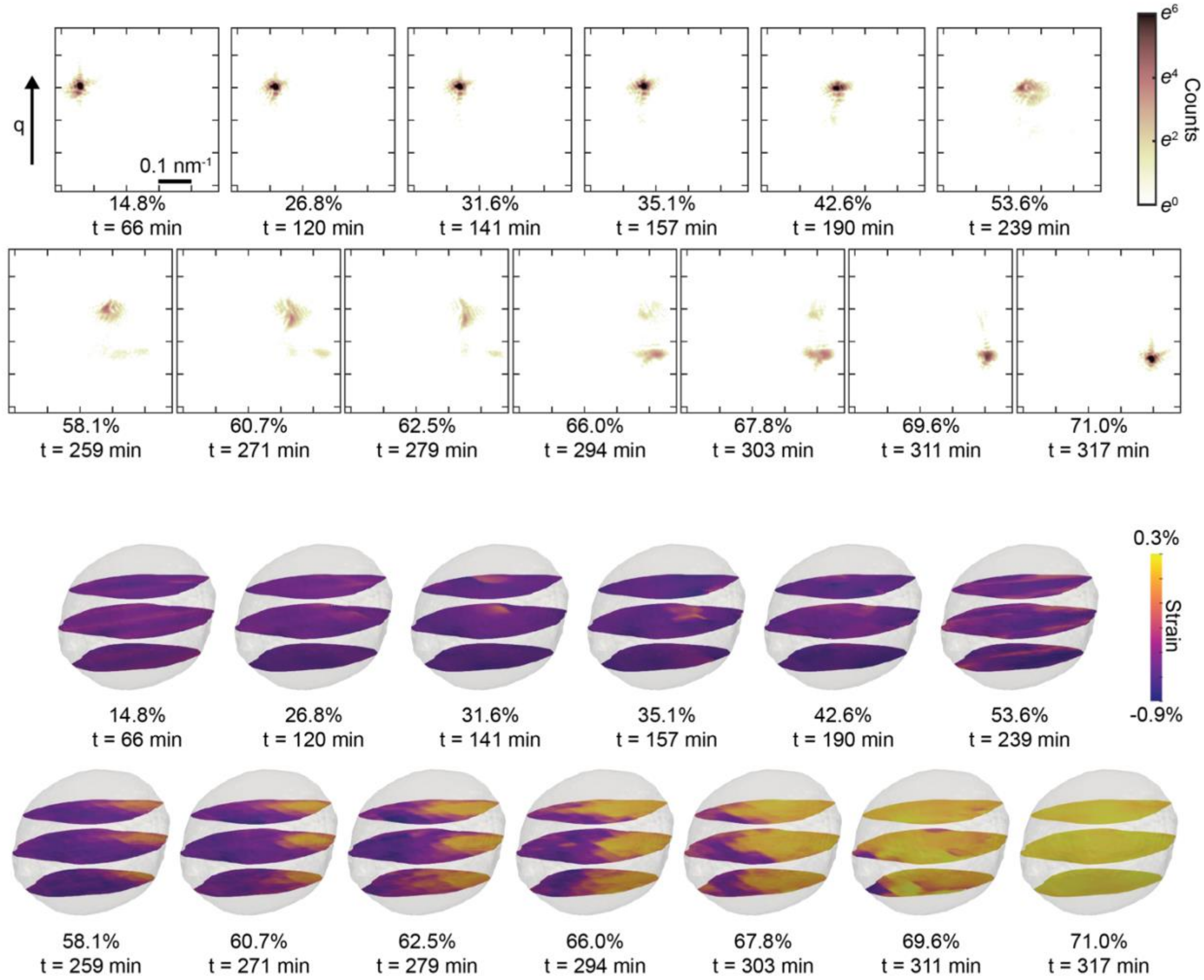

Supplementary Fig. 12. 2D diffraction slices and the corresponding inverted strain maps at three chosen planes for the supplementary particle during discharge at C/10 (18 mA/g). Similar to the primary particle discussed in the main text, this supplementary particle also exhibits a nucleation and growth regime, where the nucleated Li-rich phase grows through interface propagation. Between 26.8% DoD to 58.1% DoD, the nucleus moves and reorganizes, likely driven by surface tension reduction. The images appear less smooth due to being averaged over fewer reconstruction runs.

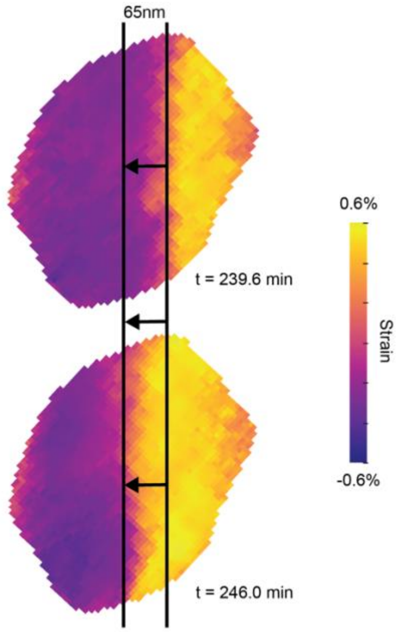

Supplementary Fig. 13. Interface velocity measured during the operando imaging experiment. Looking at specifically the region where a linear interface exists, we approximate the distance that the interface travels to be around 65 nm in 384 seconds. In three-dimensional diffusion in spinel $Li_xNi_{0.5}Mn_{1.5}O_4$, the movement of ions can be described with mean square displacement $< x^2 >= 6Dt$, where D is the diffusion coefficient and t is the time. Using bulk diffusion coefficient of Li in $Li_xNi_{0.5}Mn_{1.5}O_4$, $D_{Li} = 2 \times 10^{-12}$ cm^2/s [5], we calculate the average distance for the given time, $x = \sqrt{6Dt} = \sqrt{6 * 2 * 10^{-12}\ \mathrm{cm}^2/s * 494\ \mathrm{s}} = 6.8 \times 10^{-5}\mathrm{cm} = 680\ \mathrm{nm}$. This is one order of magnitude larger than the 65 nm we observe. We conclude that at the discharge rate, the interface propagation is not limited by Li diffusion in the particle.

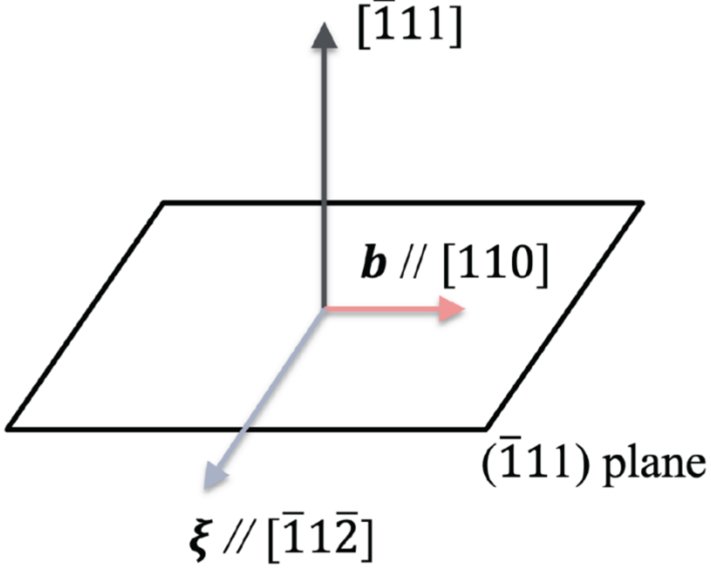

Supplementary Fig. 14. Geometry of the dislocations used in microelasticity modeling. During discharge, $Li_xNi_{0.5}Mn_{1.5}O_4$ transforms from a cubic-spinel to another cubic-spinel phase with a lattice mismatch of 0.9% (consistent with the literature and estimated directly from the diffraction data through peak splitting). The coherency strain is the same along all three principal axes of the <100> family. We find the stress tensor by multiplying the strain tensor with the elastic stiffness tensor. The elastic stiffness tensor was adopted from a similar spinel material $LiTi_2O_4$ [6] and assumed equal for both phases. For the semi-coherent interface to reflect the coherency loss along [110], we rotate the strain matrix along the z direction by 45 degrees to align [110] on [100] and set it to be 0, then transform it back to the previous coordinates.

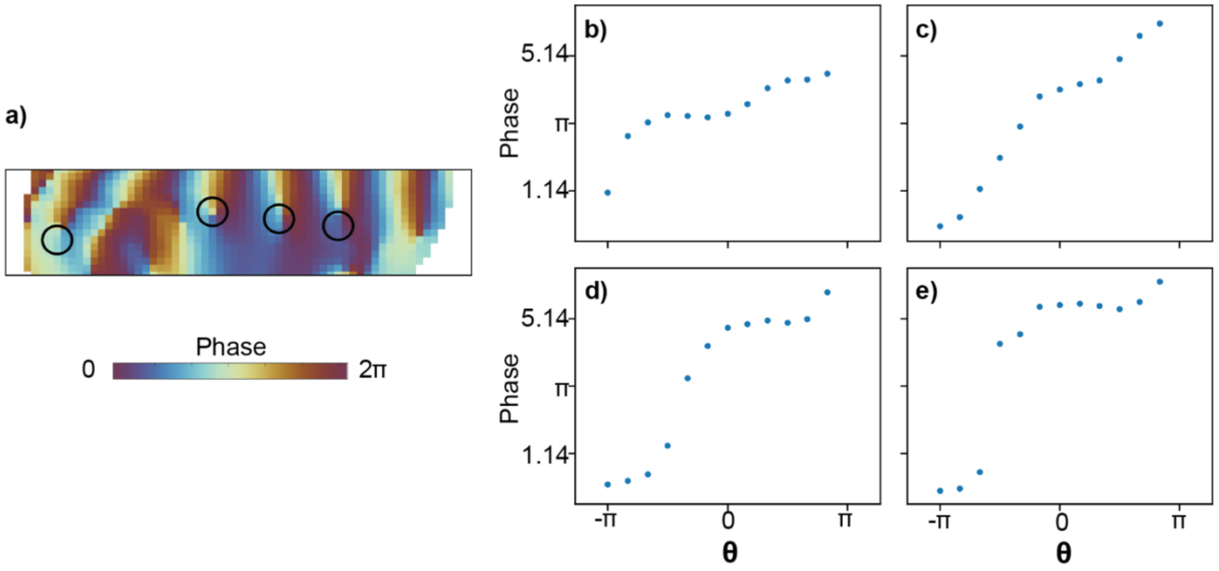

Supplementary Fig. 15. Identifying singularities and the azimuthal scans indicating dislocations. a) The enlarged phase map at 58.8% DoD same as Figure 4c. **b) - e)** The azimuthal scans around the singularities as circled in **a)**. All scans show an average phase jump of around 5 radians.

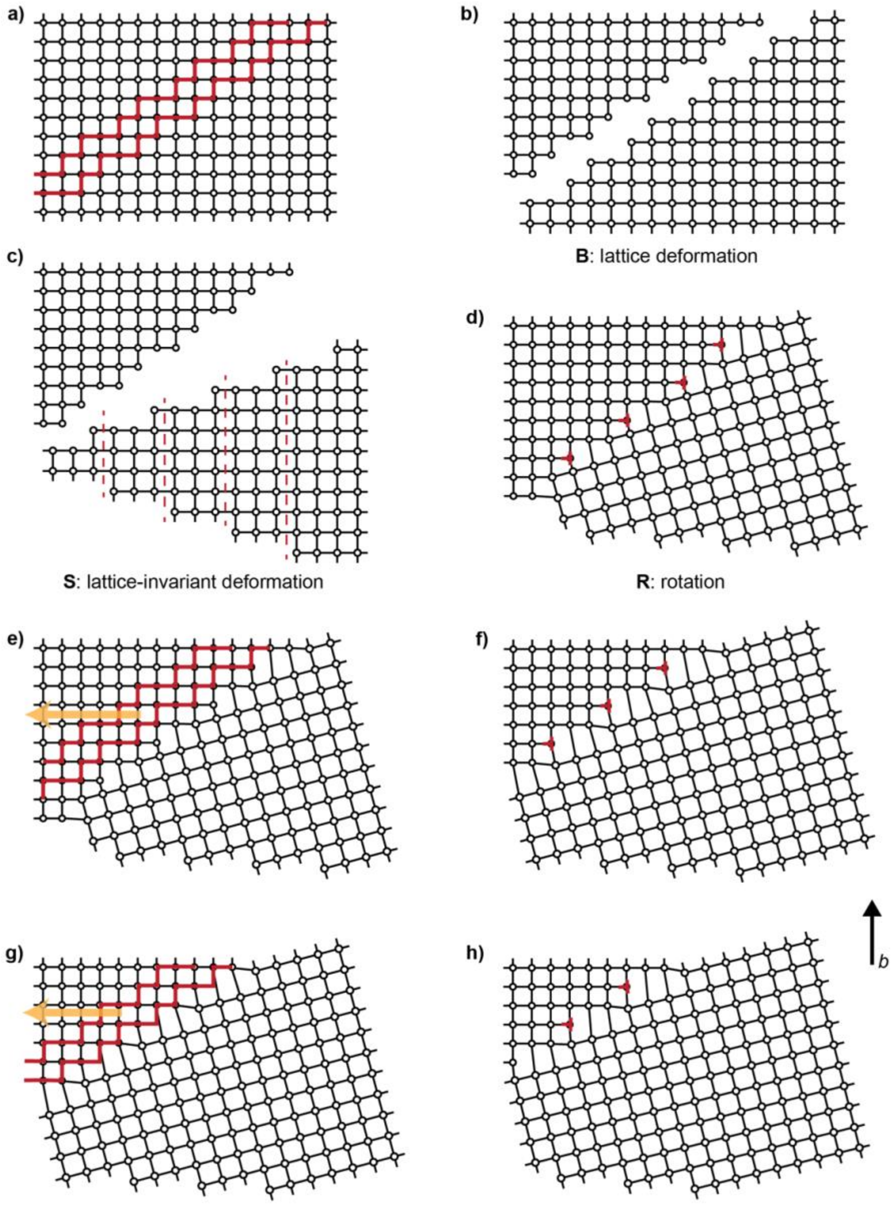

Supplementary Fig. 16. 2D illustration of the creation and propagation of a glissile interface. The example shows the growth of a square structural phase with a larger lattice parameter, similar to the growth of Li-rich $Li_xNi_{0.5}Mn_{1.5}O_4$ which has a cubic structure with a larger lattice parameter. The operation to minimize coherency strain at the interface includes lattice deformation $\boldsymbol{B}$, lattice-invariant shear deformation $\boldsymbol{S}$, and rotation $\boldsymbol{R}$[7]. The lattice-invariant deformation introduces slip with misfit dislocations at the interface, which move along the slip planes during interface propagation. Each snapshot shows an array of dislocations (**d**, **f**, **h**). During the subsequent snapshot, the dislocation array (each consisting of a different set of dislocations) moves perpendicular to the Burgers vector. This occurs via conservative motion where new dislocations enter the interface at the surface and exit the interface at the opposite surface.

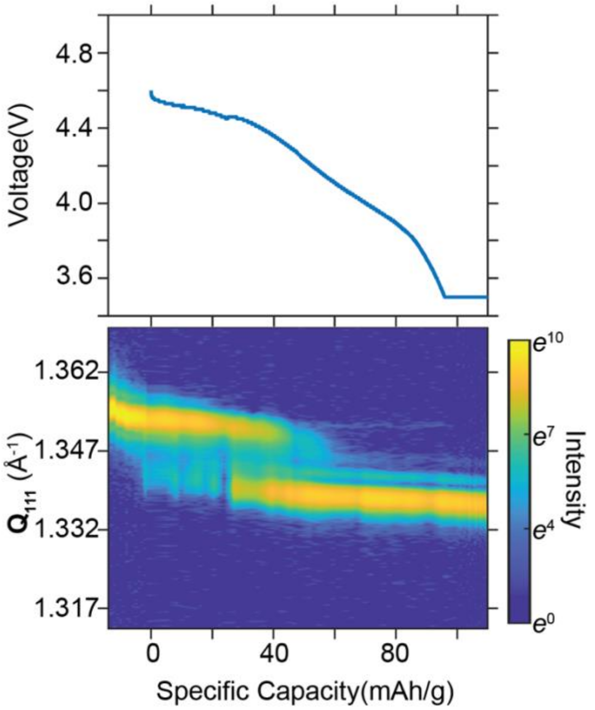

Supplementary Fig. 17. Electrochemical data of the cell (top) and diffraction data of a single $Li_xNi_{0.5}Mn_{1.5}O_4$ particle within the cell (bottom) at discharge rate of C/2 (80 mA/g). The two-phase coexistence is still present at a much higher discharge rate. This supports our hypothesis that the phase separation in $Li_xNi_{0.5}Mn_{1.5}O_4$ does not necessarily limit its kinetics as dislocations play an important role in reducing energy barriers for the reaction.

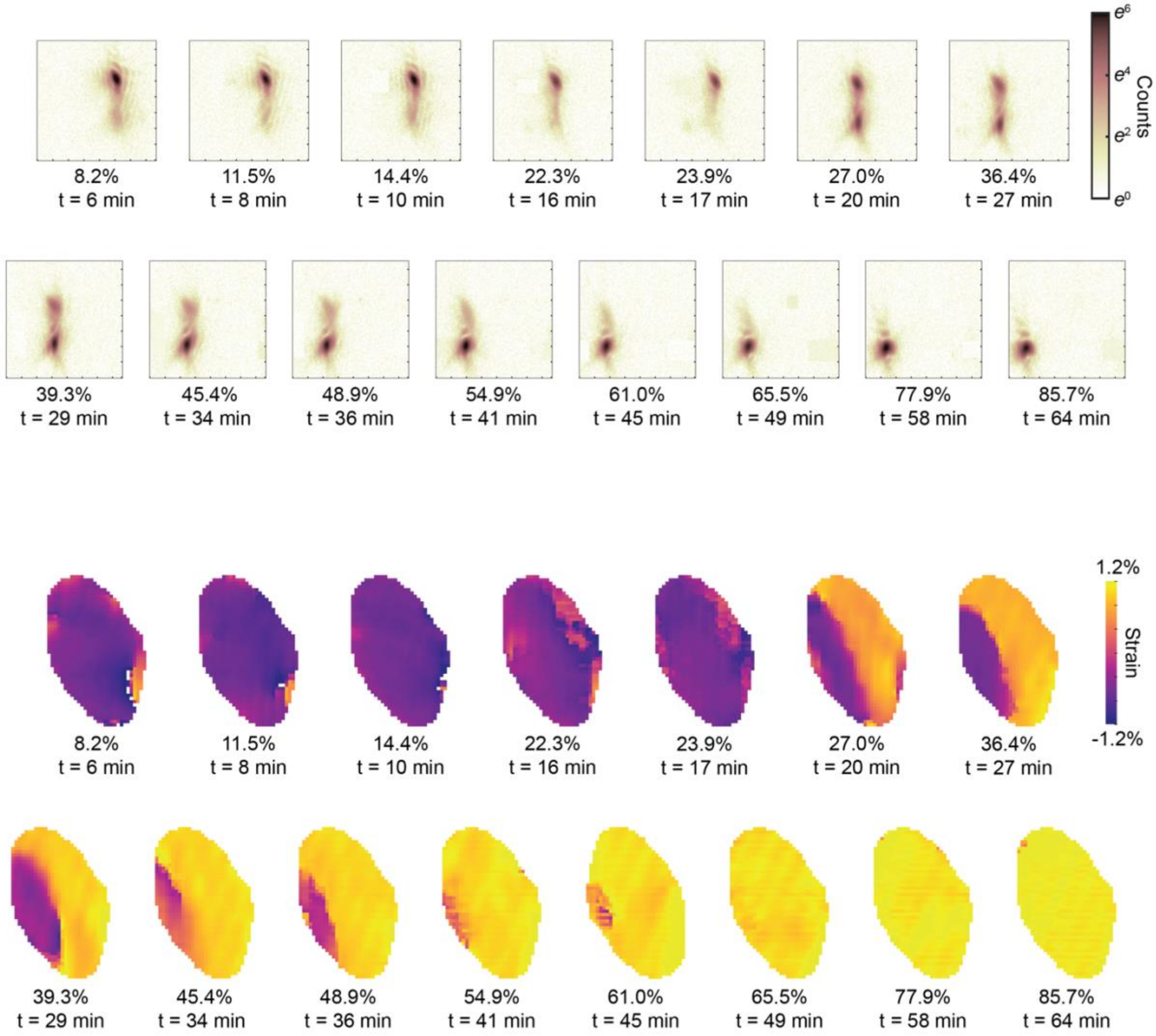

Supplementary Fig. 18. Operando imaging of the structural phase transformation during discharge at C/2 (80 mA/g). Phase retrieval of the data shown in S17 and collected at a discharge rate of C/2 (80 mA/g). Akin to a slower discharge rate of C/10 (18 mA/g), nucleation and growth are visible.

Supplementary References

1. Williams, G. J., Pfeifer, M. A., Vartanyants, I. A. & Robinson, I. K. Three-Dimensional Imaging of Microstructure in Au Nanocrystals. *Physical Review Letters* **90**, 4 (2003).
2. Fienup, J. R. Phase retrieval algorithms: a comparison. *Appl. Opt.* **21**, 2758 (1982).
3. Luke, D. R. Relaxed averaged alternating reflections for diffraction imaging. *Inverse Problems* **21**, 37 (2004).
4. Singer, A. *et al.* Nonequilibrium structural dynamics of nanoparticles in LiNi1/2Mn3/2O4 cathode under operando conditions. *Nano Letters* **14**, 5295–5300 (2014).
5. Kunduraci, M. & Amatucci, G. G. The effect of particle size and morphology on the rate capability of 4.7V LiMn1.5+δNi0.5−δO4 spinel lithium-ion battery cathodes. *Electrochimica Acta* **53**, 4193–4199 (2008).
6. Qi, Y., Hector, L. G., James, C. & Kim, K. J. Lithium Concentration Dependent Elastic Properties of Battery Electrode Materials from First Principles Calculations. *J. Electrochem. Soc.* **161**, F3010 (2014).
7. Balluffi, R. W., Allen, S. M. & Carter, W. C. *Kinetics of Materials*. (John Wiley & Sons, 2005).
